# Validation of machine learning-based models to predict and explain the risk of ovarian cancer: a multicentric study on *BRCA*-mutated patients undergoing risk-reducing salpingo-oophorectomy

**DOI:** 10.3389/fonc.2025.1574037

**Published:** 2025-04-15

**Authors:** Vera Loizzi, Maria Colomba Comes, Francesca Arezzo, Adriana Ionelia Apostol, Samantha Bove, Annarita Fanizzi, Robert Fruscio, Vanesa Gregorc, Francesco Legge, Rosanna Mancari, Claudia Marchetti, Serena Negri, Giorgia Russo, Laura Vertechy, Giovanni Scambia, Raffaella Massafra, Gennaro Cormio

**Affiliations:** ^1^ S.S.D. Ginecologia Oncologica Clinicizzata, IRCCS Istituto Tumori Giovanni Paolo II, Bari, Italy; ^2^ Dipartimento di Biomedicina Traslazionale e Neuroscienze (DiBraiN), University of Bari Aldo Moro, Bari, Italy; ^3^ Laboratorio di Biostatistica e Bioinformatica, IRCCS Istituto Tumori Giovanni Paolo II, Bari, Italy; ^4^ Dipartimento Scienze della Salute della Donna, del Bambino e di Sanità Pubblica, Fondazione Policlinico Universitario Agostino Gemelli, IRCCS, Rome, Italy; ^5^ Dipartimento Scienze della Vita e Sanità Pubblica, Università Cattolica del Sacro Cuore, Rome, Italy; ^6^ Department of Medicine and Surgery, University of Milan-Bicocca, Milan, Italy; ^7^ Division of Gynecologic Surgery, IRCCS Fondazione San Gerardo dei Tintori, Monza, Italy; ^8^ Candiolo Cancer Institute, FPO-IRCCS, Turin, Italy; ^9^ Unità di Ginecologia Oncologica, “F. Miulli” Ospedale Generale Regionale, Bari, Italy; ^10^ Gynecologic Oncology Unit, IRCCS Regina Elena National Cancer Institute, Rome, Italy; ^11^ Dipartimento Interdisciplinare di Medicina (DIM), University of Bari Aldo Moro, Bari, Italy

**Keywords:** BRCA-mutated patients, ovarian cancer risk, machine learning, artificial intelligence, risk-reducing salpingo-oophorectomy

## Abstract

**Objective:**

*BRCA*-mutated women are recommended to undergo bilateral risk-reducing salpingo-oophorectomy (RRSO) after childbearing, due to the lack of effective methods that could be able to early detect the occurrence of ovarian cancer. Thus, predictive machine learning (ML) techniques could be crucial to aid clinicians in identifying high-risk *BRCA*-mutated patients and determining the appropriate timing for performing RRSO.

**Methods:**

In this work, we addressed this task by developing explainable ML models using clinical data referred to a multicentric cohort of 694 *BRCA*-mutated patients from six Italian centers (Policlinico Gemelli, IRCCS San Gerardo, Policlinico Bari, Istituto Tumori Regina Elena, Istituto Tumori Giovanni Paolo II, Ospedale F. Miulli), who performed salpingo-oophorectomy, out of which 39 patients showed tumor (5.6%). Data from Istituto Tumori Regina Elena and Policlinico Bari were used as External Validation Cohort (EVC). The other data were employed as Investigational Cohort (IC). Resampling and ensemble techniques were implemented to handle dataset imbalance. Explainable techniques enabled us to identify some protective and risk factors predicted by the models with respect to the task under study.

**Results:**

The best ML model achieved an AUC value of 79.3% (95% CI: 75.3% - 83.0%), an accuracy value of 73.8% (95% CI: 69.6% - 78.2%), a sensitivity value of 66.7% (95% CI: 58.1% - 75.3%), a specificity value of 74.3% (95% CI: 68.7% - 80.0%), and a G-mean value of 70.4% (95% CI: 63.0% - 76.0%) on EVC. Although the model demonstrated good overall performance, its limited sensitivity reduces its effectiveness in this high-risk population. The variables CA125, age and MatoRRSO were found to be the most significant risk factors, in agreement with the clinical perspective. Conversely, variables such as Estroprogestinuse and PregnancyNfdt played a protective factor role.

**Conclusion:**

Our ML proposal explores the intricate relationships between multiple clinical variables, with a particular emphasis on understanding their non-linear associations. However, while our approach provides valuable insights into risk assessment for BRCA-mutated patients, its current predictive capacity does not significantly improve upon existing clinical models.

## Introduction

Ovarian cancer remains a challenging disease with high mortality rates, despite significant advances in medical and surgical treatment. In 2022, 19,880 new ovarian cancer cases and 12,810 related deaths emerged in the United States. According to the National Cancer Institute, ovarian cancer accounts for 1% of all newly diagnosed cancers and 2.1% of all cancer-related deaths (1). Mutations in BRCA1 and BRCA2 genes are major contributors to ovarian cancer risk, as these genes play crucial roles in DNA repair through the homologous recombination pathway, ensuring cellular genetic stability. Pathogenic mutations in these genes can lead to deficient protein function, increasing cancer risk (2). Women with BRCA1 mutations have a 35-70% lifetime risk of ovarian cancer, while BRCA2 mutation carriers have a 10-30% risk (3, 4). Ovarian cancers associated with BRCA mutations tend to be high-grade serous carcinomas, a particularly aggressive tumor type that often develops at a younger age than sporadic ovarian cancers (5). Due to the lack of effective early detection methods and the poor prognosis associated with advanced-stage ovarian cancer, bilateral risk-reducing salpingo-oophorectomy (RRSO) is recommended for BRCA mutation carriers who have completed childbearing (6). The NCCN Guidelines Panel recommends RRSO for carriers of a known BRCA1/2 variant, typically between the ages of 35 and 40 for carriers of a BRCA1 variant. Since the onset of ovarian cancer tends to occur later in carriers of a BRCA2 variant, RRSO for managing ovarian cancer risk in these women is recommended between the ages of 40 and 45, unless the age at diagnosis in the family warrants considering this prophylactic surgery at an earlier age. (NCCN Guidelines Version 2.2025) (7).

Several important studies underscore the critical timing of RRSO, such as the Normal Risk Ovarian Screening Study (NROSS) (NCT00539162) and the UK Collaborative Trial of Ovarian Cancer Screening (UKCTOCS), which highlight the need to balance early detection benefits with the timing of preventive actions (8, 9).

Recently, research works have explored machine learning (ML) algorithms as tools to aid in ovarian cancer diagnosis and inform treatment decisions (10–12). This growing interest reflects a demand for automated, personalized analyses of clinical patient data that can facilitate decision-making specific to each patient’s risk profile (13).

According to the state of the art, however, few applications of ML models to identify high-risk BRCA-mutated patients and to determine the optimal timing for RRSO have been proposed. This gap likely arises due to the relatively low incidence of ovarian cancer compared to the number of BRCA mutation carriers undergoing RRSO (14). In this vein, this study aims to develop a ML model for predicting ovarian cancer risk in BRCA-mutated patients. We expand upon our previous work (15) by applying this model to a multicenter cohort across six Italian centers, offering a broader perspective. A key aspect of the study is the use of eXplainable Artificial Intelligence (XAI) (16) to make the model’s decisions interpretable, ensuring that clinical experts can align model predictions with current clinical knowledge. By identifying and analyzing key clinical features associated with ovarian cancer risk, the study seeks to provide actionable insights for patient management and inform future clinical practice.

## Methods

### Inclusion and exclusion criteria

In this study, we included women diagnosed with BRCA1 or BRCA2 mutations who underwent bilateral RRSO at one of the six participating referred Italian centers (Policlinico Gemelli, IRCCS San Gerardo, Policlinico Bari, Istituto Tumori Regina Elena, Istituto Tumori Giovanni Paolo II, and Ospedale F. Miulli). Patients eligible for inclusion were those who had completed childbearing and were advised to undergo RRSO as part of their clinical management for ovarian cancer prevention. Only those with confirmed genetic testing results for BRCA1 or BRCA2 mutations were considered.

The inclusion criteria further required that patients had a comprehensive clinical record, including relevant demographic and medical information, and had undergone RRSO based on their BRCA mutation. Exclusion criteria included women who had not undergone RRSO or had already a diagnosis of an ovarian cancer.

A total of 694 BRCA-mutated patients met these inclusion criteria and underwent RRSO. Of them, 39 cases of ovarian or fallopian tube cancer (5.6%) were identified based on their postoperative pathology results. However, these 39 patients reported to have a malignant tumor were asymptomatic and had negative preoperative imaging.

### Study design

This study employed a retrospective multicenter design, enrolling two separate cohorts of BRCA-mutated patients who underwent bilateral RRSO. The primary aim was to develop and validate a ML model to predict the risk of ovarian cancer in BRCA mutation carriers, addressing a critical classification problem due to the imbalanced nature of the dataset. Specifically, the model was designed to classify patients into two classes: those diagnosed with ovarian cancer (rare class), and those without ovarian cancer (abundant class). The first cohort, consisting of 550 patients, served as the investigational cohort (IC) for model training and internal validation. The second cohort, comprising 144 patients, was designated as the external validation cohort (EVC) to assess the model’s generalizability to new, unseen data. The imbalanced dataset posed a significant challenge in developing an accurate ML model, as the rare class of ovarian cancer cases represented only 5.8% and 4.9% of the total patient populations in the IC and EVC cohorts, respectively. To address this issue, we applied advanced techniques in ML to enhance the prediction of the rare class. The model’s objective was to predict the ovarian cancer risk in these patients, so that it could be possible to guide decisions on the timing of RRSO. Internal validation was conducted using a leave-one-out cross-validation (LOO) scheme, where each individual sample was used as a test set exactly once, with the remaining data serving as the training set. The EVC was then employed to assess the generalizability of the model. Overall, the total study population consisted of 694 BRCA-mutated patients who met the inclusion criteria and underwent RRSO. Among them, postoperative pathological analysis identified 39 cases (5.6%) of invasive ovarian or fallopian tube cancer, as well as serous tubal intraepithelial carcinoma (STIC). Notably, However, these 39 patients reported to have a malignant tumor were asymptomatic and had negative preoperative imaging, underscoring the challenge of early detection in this high-risk population.

**Figure 1:**
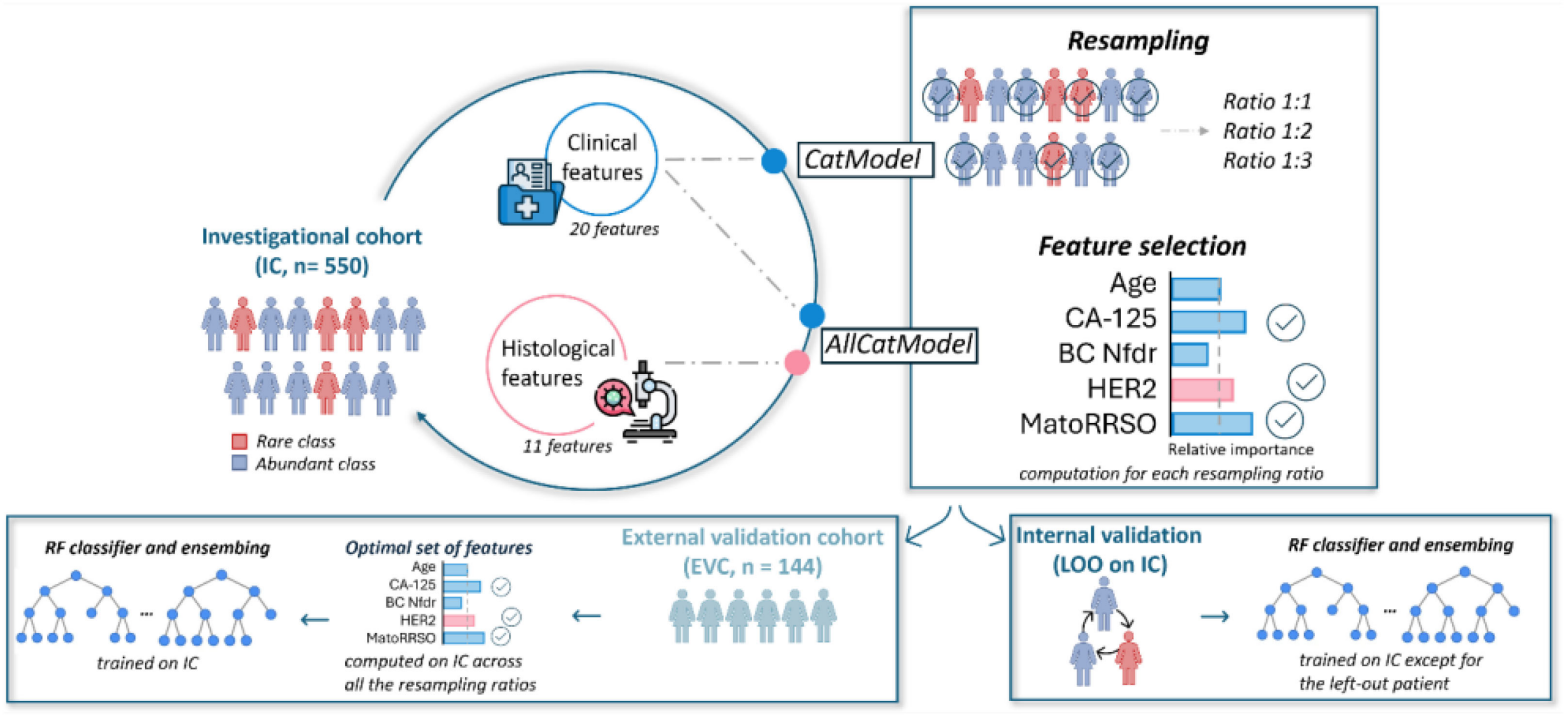
A graphical overview of the study framework. The abbreviations reported in the Figure are explained: MatoRRSO (menopause at time of RRSO), BC Nfdr (number of breast cancer first-degree relatives), LOO (leave-one-out).

A graphical overview of the study framework is provided in **Figure 1**. The study adhered to the ethical standards of the Declaration of Helsinki and was approved by the Ethics Committee of IRCCS Istituto Tumori Giovanni Paolo II, Bari, Italy (protocol code 596/CE). In accordance with the journal’s guidelines, we will provide our data for independent analysis by a selected team by the Editorial Team for the purposes of additional data analysis or for the reproducibility of this study in other centers if such is requested.

### Data collection

Clinical data for the study were retrospectively collected from medical records across six participating Italian cancer centers: Policlinico Gemelli – Rome (Gemelli), IRCCS San Gerardo – Monza (Monza), IRCCS Istituto Tumori Giovanni Paolo II – Bari (OncoBa), Ospedale F. Miulli – Acquaviva delle Fonti – Bari (Miulli), Istituto Tumori Regina Elena – Rome (Regina Elena), and Policlinico Bari (Policl. Ba). Data related to the IC were provided by the first four centers, while data for the external validation cohort EVC were gathered from the latter two centers. The information collected for this study included a total of 31 characteristics, which were divided into two main categories: 20 clinical features and 11 features related to breast cancer histology. Clinical features encompassed patient-specific details such as age, mutation type (BRCA1 or BRCA2), medical history, and RRSO-related data. Among them, estroprogestins use was included due to the reported effect on cancer risks in carriers of a known BRCA1/2 variant. Studies have shown that oral estroprogestins reduce the risk of ovarian cancer by 45% to 50% in BRCA1 variant carriers and by 60% in BRCA2 variant carriers. Moreover, the protective effect appears to increase with longer durations of oral contraceptive use, although the long-term benefit diminishes following menopausal hormone replacement therapy after bilateral salpingo-oophorectomy. Oral contraceptives may be considered for ovulation suppression and are not contraindicated for birth control purposes in these patients. The breast cancer histology features provided additional context relevant to the patient’s oncological profile. A comprehensive list of these features, along with their respective abbreviations, is provided in **Supplementary File 1**. To ensure the quality and reliability of the data, the clinical teams affiliated with each participating center were responsible for collecting and verifying the information. Data were cross-checked within each center for transcription accuracy and logical consistency. The finalized dataset was then reviewed by the coordinating clinical team at the lead institution, which ensured adherence to predefined inclusion and exclusion criteria. Statistical analyses were conducted to identify any inconsistencies or outliers, which were addressed through consultation with the contributing clinicians from the respective centers. This rigorous validation process ensured the integrity of the multicenter dataset used for the development and validation of the machine learning model. **Supplementary Figure 1** illustrates the distribution of enrolled patients across these centers, showing the numbers in both the rare and abundant classes for each cohort.

### Machine learning model

The dataset used was imbalanced, with fewer patients in the rare class (patients with ovarian cancer) than in the abundant class (patients without ovarian cancer). To address this, a ML model was constructed using a Random Forest classifier with resampling and ensemble techniques, both of which enhance model performance on imbalanced datasets by ensuring better representation of the rare class and aggregating strengths from multiple models to improve prediction accuracy (17–19). Specifically, we developed two types of ML models with the same architecture but differing in the input features used: the first model, named *AllCatModel*, utilized all 31 characteristics listed in **Supplementary File 1**. The second model, named *CatModel*, used the same characteristics with the exception of those related to breast cancer histology, resulting in a final set of 20 features.

#### Key concepts in the ML model

##### Resampling Technique

The model development involved resampling, creating multiple models with varied ratios of samples from the rare and abundant classes. Models were generated with ratios of 1:1, 1:2, and 1:3, ensuring that each rare class sample had one, two, or three samples from the abundant class. This approach provided balanced representation during training, thus enhancing the model’s capacity to accurately identify cases of ovarian cancer.

##### Ensemble Technique

The ensemble method combined predictions from multiple models to improve overall accuracy. Each patient’s final classification was based on predictions from models trained on different resampling ratios, with a unified prediction derived through aggregation. This technique capitalizes on diverse model outputs, creating a stronger, more reliable final prediction.

##### Model structure

The model was structured in two primary phases: feature selection and classification, both implemented using the Random Forest algorithm with 100 trees (20).

##### Feature Selection

The algorithm evaluated feature importance via Gini impurity, a metric that assesses how well a feature splits the data. Features with weights above the median were retained for classification.

##### Classification

In this phase, the classifier processed the selected features, assigning a score from 0 to 1 to each patient based on ovarian cancer risk, with higher scores suggesting a higher likelihood of ovarian cancer.

##### Workflow

**Figure 2:**
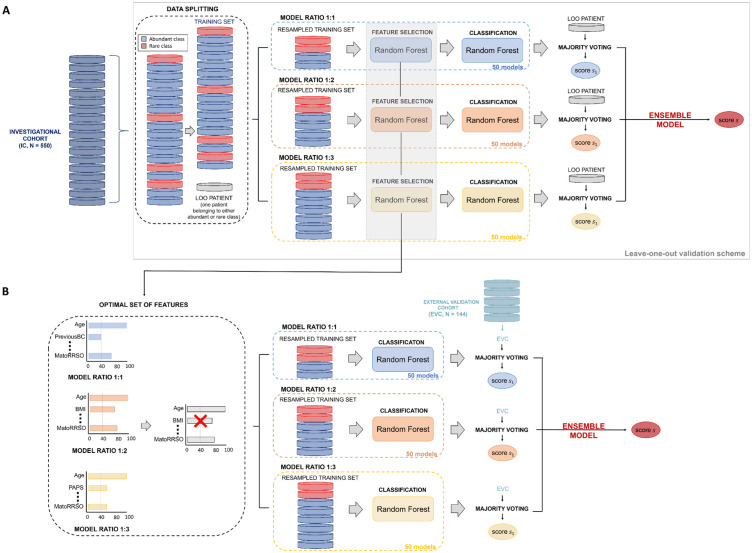
Workflow of the ML model. **(A)** Leave-one-out validation scheme on the Investigational cohort (IC).To estimate the classification score of each leaved-out patient of the training set, 50 models per ratio (ratio 1:1, ratio 1:2, ratio 1:3) were defined. Their responses were firstly joined together by majority voting and then merged by soft voting. **(B)** Validation on the External Validation Cohort (EVC). To predict the label of each sample of the validation set, 50 models per ratio (ratio 1:1, ratio 1:2, ratio 1:3) exploiting the features selected over the models on the training set with a frequency higher or equal than 40% were developed. Their responses were firstly joined together by majority voting and then merged by soft voting.

**Figures 2A, B** illustrate the ML model’s workflow and its evaluation using the IC and EVC cohorts. Internal validation was conducted with a leave-one-out (LOO) validation scheme on the IC, while external validation was performed on the EVC.

##### Internal Validation

To internally validate the model on the IC, we used a leave-one-out (LOO) scheme, where each patient served as a test sample in turn. The remaining patients were resampled in 1:1, 1:2, and 1:3 ratios to create training sets. For each excluded patient, we trained 150 models (50 for each ratio), performing feature selection and constructing a Random Forest classifier that predicted the patient’s cancer risk. A majority voting system combined predictions across models, averaging results across the three ratios to generate a final score for each patient (soft voting).

##### External Validation

Optimal feature selection was crucial for clinical application. We determined the optimal set by calculating the frequency with which each feature was selected as important in models trained with different resampling ratios on the IC. Features that were consistently selected at least 40% of the time were included in this set, and the 50 models for each ratio were retrained with these features. This ensemble model was then applied to the EVC, generating a unique classification score for each patient.

#### Statistical analysis and performance evaluation

We assessed the association between each feature and the outcome (abundant vs. rare class) using statistical tests. The Wilcoxon-Mann-Whitney test was applied for continuous features, and the Chi-Squared test was used for ordinal clinical characteristics, considering p-values below 0.05 as significant (21, 22). The model’s performance was evaluated with both ranking and threshold metrics: the Area Under the Curve (AUC) was used as a ranking metric, while accuracy, sensitivity, specificity, F1-score, and G-mean were applied as threshold metrics. These metrics were calculated after defining the optimal threshold using Youden’s index on the ROC curve (23), as follows:


Accuracy=(TP+TN)/(TP+TN+FP+FN)

Sensitivity=TP/(TP+FN)

Specificity=TN/(TN+FP)

F1−score=2∗(Sensitivity∗Precision)/(Sensitivity+Precision)

$$\text{G-mean}=\sqrt{Sensitivity*Specificity}.$$

where TP and TN represent True Positives and True Negatives, respectively, and FP and FN denote False Positives and False Negatives. The F1-score balances precision and sensitivity, while the G-mean is beneficial for evaluating imbalanced datasets due to its ability to measure performance across both classes evenly (24). Additionally, to estimate the 95% Confidence Intervals (CIs) for the model’s performance metrics, we applied bootstrap resampling with 1000 iterations. Source codes were implemented in MATLAB R2022a (MathWorks, Natick, MA, USA).

### Explainability technique

To interpret the predictions of the ML models (AllCatModel and CatModel), we used an explainability approach based on SHapley Additive exPlanations (SHAP) values (25). SHAP values quantify each feature’s contribution to the model’s predictions, with positive values indicating risk-enhancing contributions and negative values suggesting non-risk contributions. SHAP values for each feature reflect its importance to the prediction, considering interactions with other features as per game theory principles. Calculations were performed with a local-agnostic algorithm, creating a linear interpretative model for each test patient by analyzing only the classifier’s input and output. SHAP value computations were implemented using a ColabPro Notebook with Python programming (26).

## Results

### Investigational and external validation cohorts

The characteristics collected for both the IC and EVC cohorts are summarized in **Supplementary Table 1**. were statistically compared to assess their representativeness of the same underlying population. Given the imbalanced distribution of ovarian cancer cases (the rare class) and non-cancer cases (the abundant class), the IC cohort had a rare class distribution of 5.8%, while the EVC cohort had a rare class distribution of 4.9%. Statistical tests, including tests for equivalence of proportions and continuous variables, were performed to detect potential biases between the cohorts: Wilcoxon-Mann-Whitney test was performed for continuous features, whereas Chi Squared test was used for the clinical characteristics. These tests confirmed that the two cohorts were similar, minimizing discrepancies and ensuring that the ML model’s performance metrics accurately reflected its generalizability to new data. The p-values for each variable were reported in **Supplementary Table 2**.

### Feature importance

In our study, we first conducted univariate statistical tests to examine the relationship between each clinical factor and the outcome, using both the training and validation datasets (*see* Methods, Statistical analysis and performance evaluation). Only Age (p-value = 0.004) in the IC and CA125 (p-value = 0.02) in the EVC showed significant associations with the outcome. These univariate tests highlight the individual importance of each feature. To assess feature value within the ML model, a more sophisticated feature selection technique was employed. This method evaluates the relative importance of features across the entire set used in the ML model.

**Supplementary Table 3** shows the frequency with which each feature was identified as important across the 50 models at each resampling ratio (1:1, 1:2, and 1:3) for both *CatModel* and *AllCatModel*. Three features—Age, menopause at time of RRSO (MatoRRSO), and number of breast cancer first-degree relatives (BCNfdr)—were frequently selected (over 50% of the time) across all resampling ratios for both models. Features chosen at least 40% of the time were included in the optimal subset used for validation. **Supplementary Table 4** summarizes the optimal features for *CatModel* and *AllCatModel*. Common features include CA125, Age, number of pregnancy normal full term delivery (Pregnancynftd), MatoRRSO, *BRCA2*, status of ovarian cancer first-degree relatives (OCFDR), number of ovarian cancer first-degree relatives (OCNfdr), number of ovarian cancer second-degree relatives (OCNsdr), and BCNfdr. Unique features for *CatModel* were previous abdominal/pelvic surgery (PAPS) and *BRCA1*, while *AllCatModel* included Estroprogestin use and previous breast cancer (PreviousBC). Features related to breast histology—HER2, grading (Grade), invasive ductal carcinoma (IDC), and invasive lobular carcinoma (ILC)—were also part of the optimal set. Comparison with our previously published model (10) revealed that Age, Pregnancynftd, and CA125 were consistently important across both models.

### Performance evaluation and explanation

**Figure 3:**
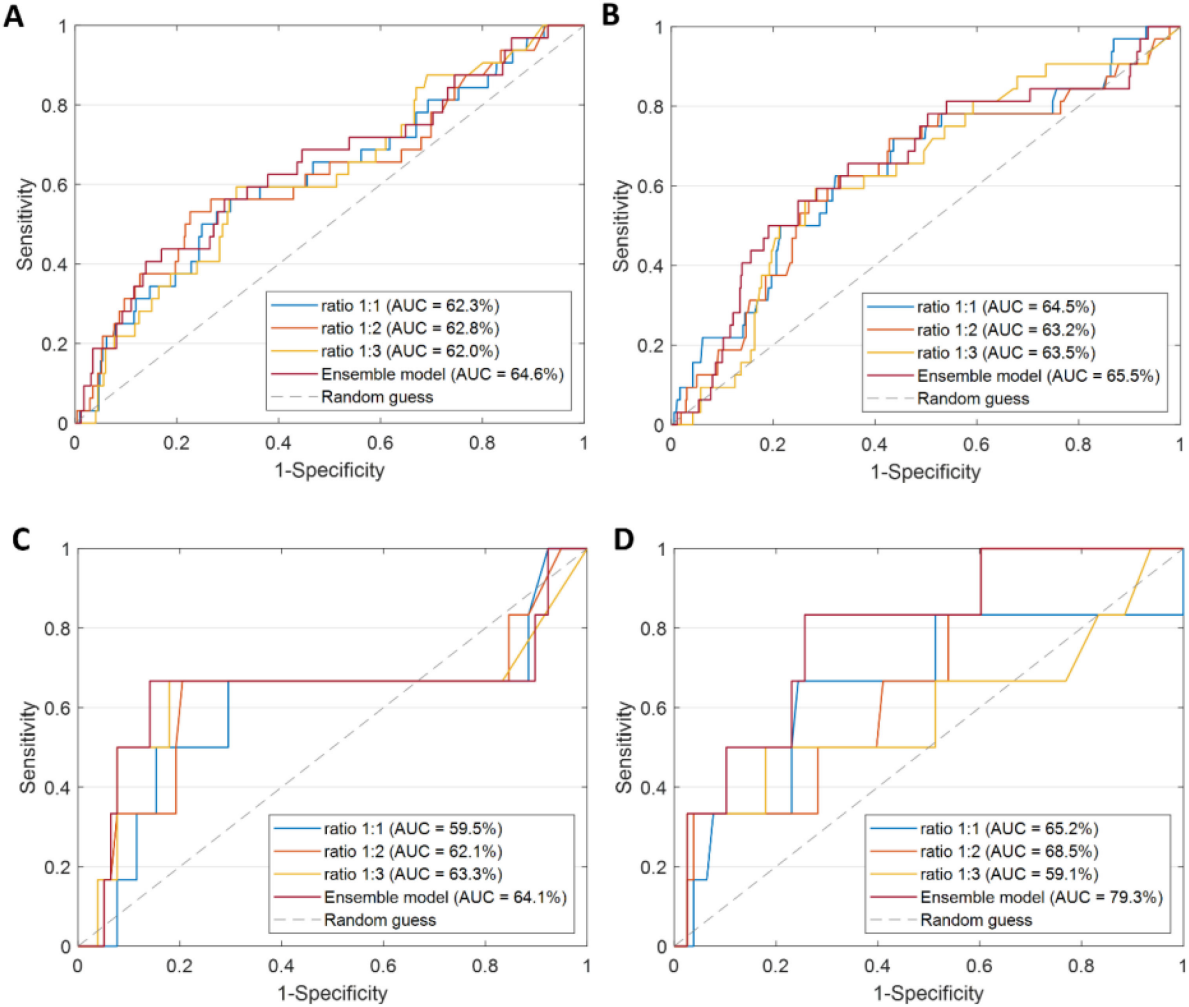
Comparison of ROC curves and the resulting AUC values. **(A, B)** *AllCatModel* and *CatModel* in LOO scheme over the training set, respectively. **(C, D)** *AllCatModel* and *CatModel* over the validation set, respectively. **(A-D)** TheROC curves of the models obtained after applying majority voting for each of the three ratios were compared with the ROC curve of the Ensemble model.

We performed a two-level validation of *AllCatModel* and *CatModel*. Initially, we assessed their performance using a leave-one-out (LOO) cross-validation scheme on the IC. The features used for classification varied across patients and sub-models within the final ensemble models. In this case, *AllCatModel* and *CatModel* achieved comparable performance in terms of all the evaluation metrics (*see* **Table 1**). The ROC curves for these final ensemble models are shown in **Figures 3A, B**. ROC curves for various resampling ratios (1:1, 1:2, 1:3) are also depicted, with AUC values of 62.3% (95% CI: 59.3% - 65.3%), 62.8% (95% CI: 59.8% - 65.8%), and 62.0% (95% CI: 58.9% - 65.0%) for *AllCatModel* and 64.5% (95% CI: 61.5% - 67.5%), 63.2% (95% CI: 60.2% - 66.2%), and 63.5% (95% CI: 60.5% - 66.5%) for *CatModel*, respectively.

**Table 1:** Performance evaluation of the two ML models, i.e., *AllCatModel* and *CatModel*.

Evaluation set	Feature set	Metric (%)	Model type	
			AllCatModel	CatModel
Investigational Cohort (LOO)	Feature selected over each model at diverse ratio	AUC	64.6 [60.0, 69.2]	65.5 [61.0, 70.0]
		Accuracy	69.6 [65.0, 73.5]	70.7 [66.0, 74.6]
		Sensitivity	53.1 [45.0, 61.0]	56.3 [48.4, 64.0]
		Specificity	70.7 [64.0, 76.1]	71.6 [65.0, 77.0]
		F1-score	29.8 [25.3, 35.0]	39.9 [33.4, 46.7]
		G-mean	61.2 [55.0, 67.6]	63.5 [57.0, 69.0]
External Validation Cohort	Optimal feature set	AUC	64.1 [59.2, 69.0]	79.3 [75.3, 83.0]
		Accuracy	79.8 [74.5, 84.0]	73.8 [69.6, 78.2]
		Sensitivity	50.0 [40.0, 60.0]	66.7 [58.1, 75.3]
		Specificity	82.1[78.2, 86.0]	74.3 [68.7, 80.0]
		F1-score	30.0 [25.0, 35.5]	44.4 [37.7, 51.5]
		G-mean	64.1 [58.3, 70.2]	70.4 [63.0, 76.0]

The evaluation set as well as the feature set adopted for the evaluation are reported. 95% Confidence Intervals (CI) for each metric are also included.

Subsequently, we evaluated the models on the EVC using an optimal subset of features. Conversely to LOO validation, the *CatModel* overall outperformed *AllCatModel*. **Figures 2C, D** shows the corresponding ROC curves, alongside the ROC curves of the models at diverse ratio with AUC values equal to 59.5% (95% CI: 56.5% - 62.5%) and 65.2% (95% CI: 62.2% - 68.2%) for ratio 1:1, 62.1% (95% CI: 59.1% - 65.1%) and 68.5% (95% CI: 65.5% - 71.5%) for ratio 1:2, 63.3% (95% CI: 60.3% - 66.3%) and 59.1% (95% CI: 56.1% - 62.1%) are obtained for ratio 1:3, in correspondence of *AllCatModel* and *CatModel*, respectively.

**Figure 4:**
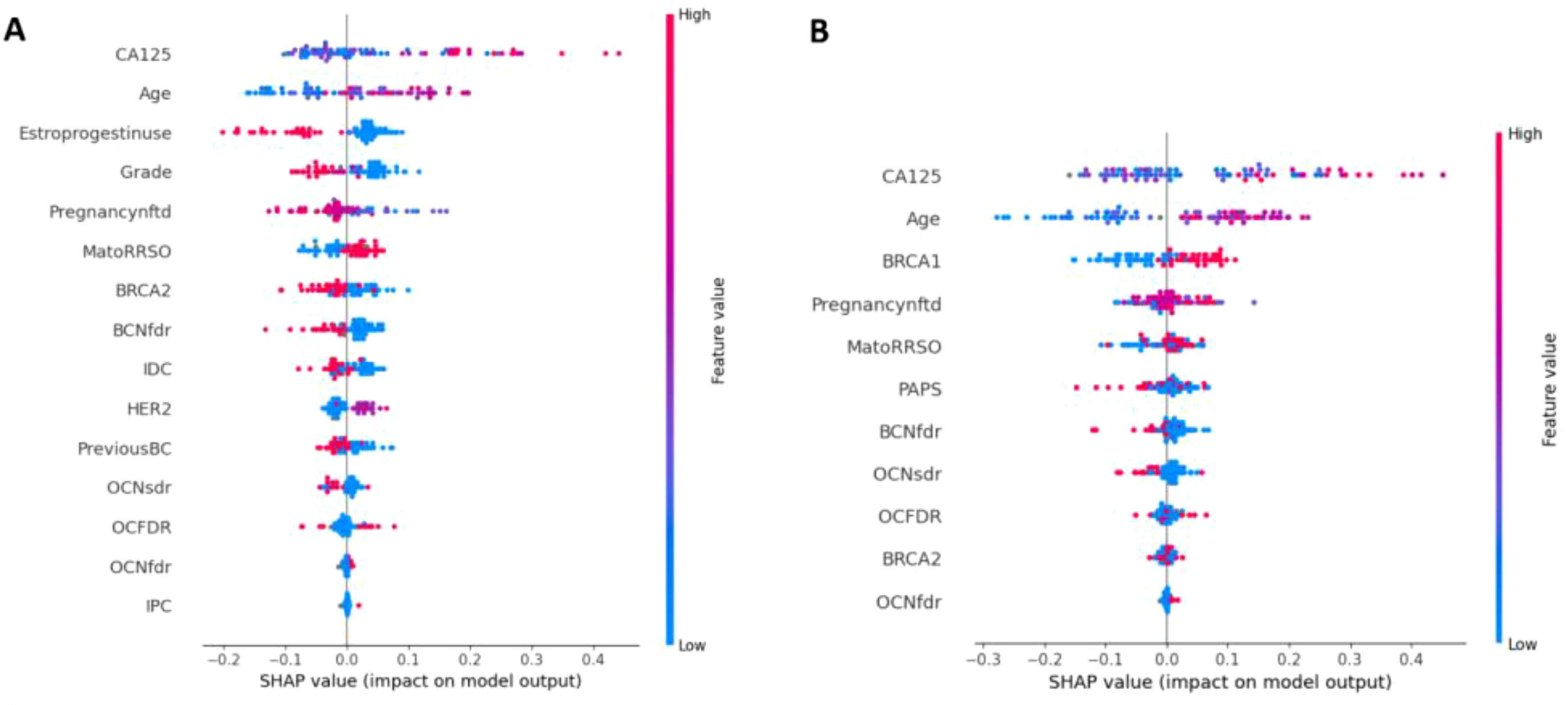
Shapley value distributions for **(A)** *AllCatModel* and **(B)** *CatModel*. Each point on the plot is a SHAP value for a feature and a patient. The color bar represents the value of the feature from low (in blue) to high (in red) for that instance. The abbreviations of all the features are summarized in **Supplementary File 1**.

**Figure 4** depicts the features that most influenced the classification scores (**Figure 4A** for *AllCatModel*, **Figure 4B** for *CatModel*, respectively). Features are ranked in descending order according to their relative importance computed by the SHAP algorithm. Each point represents the Shapley value for a feature and a patient of the validation set. The relationship between a higher or lower feature value and a higher or lower prediction (classification score for the rare class) is also highlighted. In this way, we were able to visualize some protective and risk factors predicted by the models with respect to the task under study: features whose higher values contribute to the outcome occurrence were considered as risk factors (red points for positive SHAP values, blue points for negative SHAP values, respectively); features whose higher values go against the outcome occurrence were treated as protective factors (blue points for positive SHAP values, red points for negative SHAP values, respectively). Similarly to feature importance, since the importance of a feature was computed by evaluating the relative importance of the features with respect to all the features involved in the two models, as well as the *AllCatModel* involved breast histology features not included in the *CatModel*, features that were deemed as important for one model not necessarily had the same importance for the other model.

Concerning *AllCatModel*, the variables CA125, age and MatoRRSO were found to be the most significant risk factors, in agreement with the clinical perspective. Conversely, variables such as Estroprogestinuse and PregnancyNfdt played a protective factor role. With respect to *CatModel*, the variables CA125, age, and *BRCA1* were revealed as the most remarkable risk factors.

## Discussion

In this study, we developed a ML model to identify BRCA-mutated patients at high risk of ovarian cancer, aiding in determining optimal timing for RRSO. The model, trained and validated on clinical data from a multicenter cohort across six Italian cancer centers, builds on our previous work, where we initially developed a hypothesis-generating method for this clinical challenge. The analysis utilized two cohorts: IC and EVC. As evidenced by both the statistical analysis comparing the two cohorts and the analysis within each cohort in relation to the outcome, no selection bias was identified between the cohorts, ensuring the robustness and generalizability of the model. By expanding our study to a multicenter setting, we confirmed the model’s predictive ability, demonstrating its broader feasibility and potential for larger-scale use.

One of this study’s major contributions is the identification and validation of clinical features impacting the ML model’s predictions. The study includes a large, multicenter cohort and the use of explainable machine learning techniques have provided valuable insights into the key clinical variables associated with ovarian cancer risk in BRCA-mutated patients. We analyzed how the model classified each feature, distinguishing between those identified as risk and protective factors. This process was further strengthened by a multidisciplinary collaboration, which ensured that model insights aligned with clinical knowledge and create a bridge between data interpretation and clinical expertise. This collaborative approach enhances the clinical relevance and potential application of the model in real-world settings.

Compared to other recently developed ML models applied in ovarian cancer care, our study tackles a highly specific and challenging context: the prediction of ovarian cancer risk in BRCA-mutated patients considering prophylactic surgery. For example, Ledger et al. (27) evaluated six machine learning algorithms to estimate the probability that ovarian tumors are benign, borderline malignant, or primary invasive, achieving AUC values ranging from 76.0 to 85.0%. Similarly, He et al. (28) compared the diagnostic efficacy of machine learning models with expert subjective assessment in evaluating the malignancy risk of ovarian tumors, achieving an AUC of 89.0%. Our model, in contrast, focuses exclusively on BRCA-mutated patients and addresses a significantly imbalanced dataset. This imbalance poses additional challenges, as predictive models must balance sensitivity and specificity under these constraints. Despite this, our model achieved an AUC of 79.3% on the external validation cohort, comparable to some models in the broader literature.

The ML analysis identified CA125 levels, patient age, and MatoRRSO as primary risk factors for ovarian and fallopian tube cancers in BRCA1 and BRCA2 mutation carriers, consistent with current clinical understanding. Conversely, features such as estroprogestin contraception use and the number of full-term pregnancies (PregnancyNfdt) appeared as protective factors in the model’s predictions. Notably, BRCA-related ovarian cancers often develop without a family history of breast or ovarian cancer—about 40% of patients with ovarian or fallopian tube cancer who are BRCA mutation carriers lack this family history. This finding underscores the value of genetic testing for relatives, enabling first- and second-degree relatives to assess their own cancer risk and consider preventive measures (29, 30).

Genetic screening is crucial, especially given variations in peak risk ages among BRCA1/2 carriers. BRCA1 mutation carriers have the highest risk of ovarian cancer between ages 50 and 59, with an annual incidence of 1.7%, whereas BRCA2 mutation carriers peak later, between 60 and 69, with an annual incidence of 0.6% (6, 31). A retrospective cohort study of 474 BRCA1/2 carriers with high-grade serous ovarian cancer also revealed a significantly higher average age at diagnosis for BRCA2 versus BRCA1 mutation carriers (58.4 years vs. 53.3 years, P = .001) (6, 32).

Effective screening strategies remain a topic of ongoing research. CA125 monitoring and transvaginal ultrasound (TVUS) have been explored for early cancer detection in BRCA mutation carriers but show variable sensitivity and specificity. The UK Collaborative Trial of Ovarian Cancer Screening (UKCTOCS) compared multimodal screening (CA125 with TVUS) to TVUS alone or no screening. While combining TVUS and CA125 improved early-stage detection, there was no significant reduction in mortality after a median follow-up of 11 years (7, 33). Moreover, CA125 levels can be elevated due to benign conditions or other malignancies, which complicates interpretation, as high CA125 levels without abnormal ultrasound findings may indicate occult ovarian or tubal cancer (8, 34, 35).

For BRCA mutation carriers, estrogen-progestin contraception may reduce ovarian cancer risk by suppressing ovulation, serving as a protective factor while awaiting RRSO. Similarly, pregnancy suppresses ovulation and is protective against ovarian cancer in high-risk populations (16, 36).

Despite its promising outcomes, the model developed in this study is not yet ready for clinical use due to its limited sensitivity (66.7%). While the model shows good accuracy in distinguishing risk and protective factors, the sensitivity remains a limitation that may reduce its clinical utility in detecting all high-risk patients. Additionally, the model’s performance may be influenced by the quality and consistency of the clinical data from different centers, highlighting the need for further validation and refinement. Moreover, the study is limited to Italian centers, which may affect the generalizability of the results to other populations or healthcare systems. Future work should focus on improving model performance and developing an interface to assist clinicians in identifying high-risk BRCA patients and determining RRSO timing. Additionally, separate machine learning models for BRCA-1 and BRCA-2 mutations will be explored to assess whether this approach improves prediction accuracy by creating more homogeneous groups, thereby enabling more tailored and precise risk assessments for BRCA-mutated patients. Finally, although lifestyle factors such as smoking, which has been associated with ovarian cancer risk (37), were not considered in this study. Incorporating these factors into future models could improve the overall prediction accuracy and provide a more comprehensive understanding of ovarian cancer risk in BRCA-mutated patients. Therefore, while the current work provides a valuable foundation, further refinement of the model is necessary to enable a personalized and accurate risk assessment, as it does not yet offer a more tailored approach than the existing clinical strategies.

## Conclusions

This study represents a first effort toward supporting clinicians in risk stratification for BRCA mutation carriers. Current guidelines recommend prophylactic adnexectomy for BRCA1/2 carriers at specific ages, but early-onset ovarian cancer remains a possibility, and many patients may never develop cancer. This underscores the need for a nuanced approach to risk assessment. Our model leverages non-linear relationships among patient variables, offering a more personalized risk evaluation. Moving forward, further development is needed, including performance optimization, integration into clinical workflows, and validation through prospective clinical trials to assess its real-world impact on patient outcomes.

## Data Availability

The raw data supporting the conclusions of this article will be made available by the authors, without undue reservation.

## Supplementary material

Supplementary Figure 1 Overview on the multicentric data collection. Histogram of the number of patients enrolled across the Italian centers. The number of patients belonging to both the abundant and rare classes are also indicated.

Supplementary File 1 List of clinical and histological characteristics, their abbreviations and corresponding values.

Supplementary Table 1 Clinical characteristics of both Investigational Cohort (IC) and External Validation cohort (EVC). For categorical variables, absolute and percentage counts are reported in round brackets. For continuous variables, the median value and first (q_1_) and third (q_3_) quartiles of the distribution are indicated in squared brackets. The number of missing values (NA) is also specified.

Supplementary Table 2 Summary of p-Values from Statistical Tests Comparing Clinical Data of the Investigational Cohort (IC) and External Validation Cohort (EVC). A result was considered statistically significant when the p-value was less than 0.05.

Supplementary Table 3 Feature selection for **(A)** *AllCatModel* and **(B)** *CatModel* on the training set. Frequency of the features in all the diverse ratio models have been reported. Frequencies greater than 40% are highlighted in bold. The abbreviations of all the features are summarized in **Supplementary File 1**.

Supplementary Table 4 Overview on the optimal feature set. Features selected with frequency higher or equal than 40% for all the model ratios were reported in each column.
