# Supplementary figures and images for: Validation of machine learning-based models to predict and explain the risk of ovarian cancer: a multicentric study on BRCA-mutated patients undergoing risk-reducing salpingo-oophorectomy
Source: Front Oncol. 2025 Apr 15;15:1574037. doi: 10.3389/fonc.2025.1574037 (PMC12037974; doi:10.3389/fonc.2025.1574037)

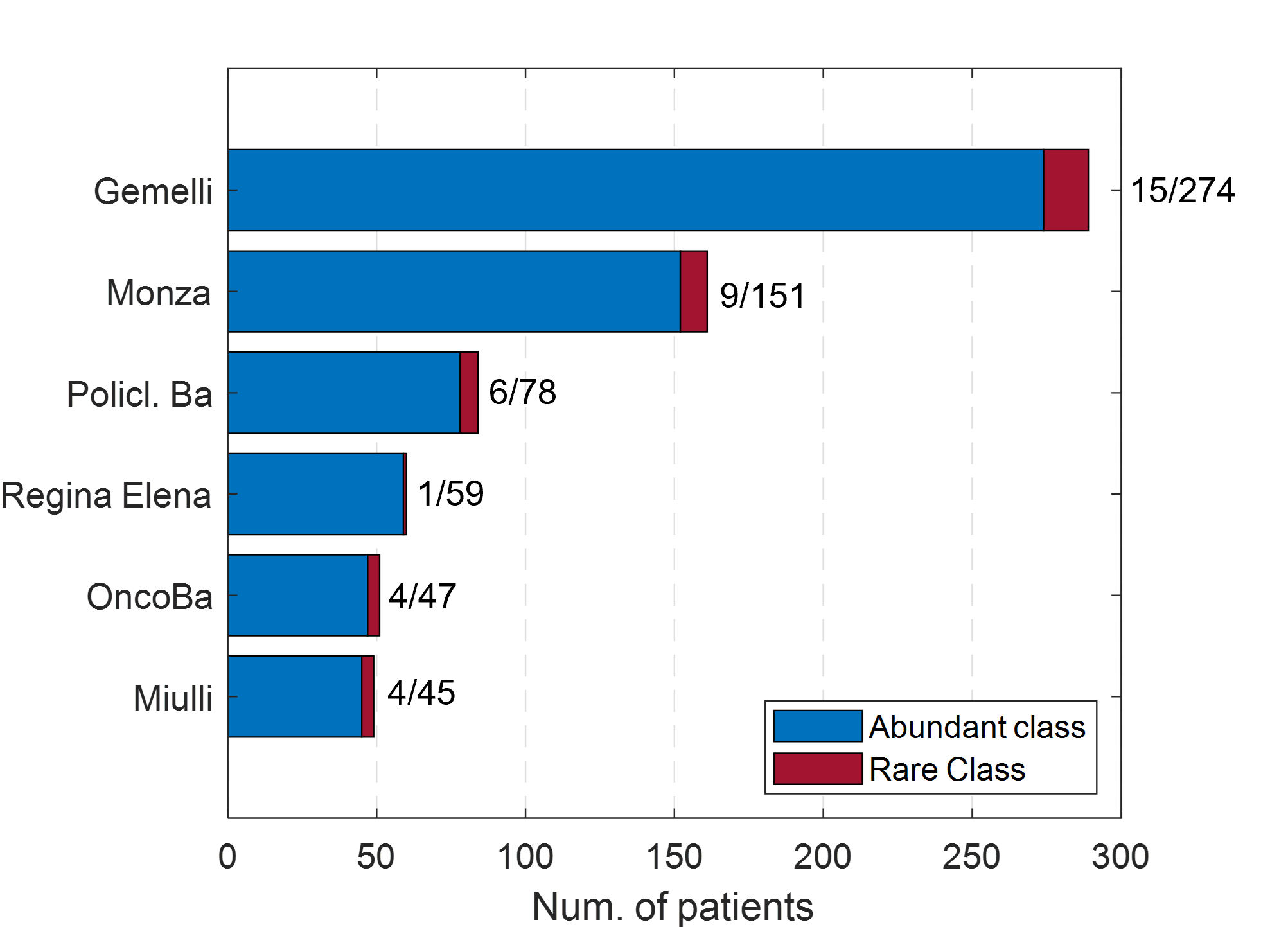

Supplement: Supplementary Figure 1 — Overview on the multicentric data collection. Histogram of the number of patients enrolled across the Italian centers. The number of patients belonging to both the abundant and rare classes are also indicated. [file Image1.tif]
