# Supplementary material for: Validation of machine learning-based models to predict and explain the risk of ovarian cancer: a multicentric study on BRCA-mutated patients undergoing risk-reducing salpingo-oophorectomy
Source: Front Oncol. 2025 Apr 15;15:1574037. doi: 10.3389/fonc.2025.1574037 (PMC12037974; doi:10.3389/fonc.2025.1574037)
Supplement: Supplementary File 1 — List of clinical and histological characteristics, their abbreviations and corresponding values. [file DataSheet1.docx]

**Supplementary file 1**

The collected clinical characteristics comprised both categorical and continuous variables. In the following, there is the list of clinical and histological characteristics their abbreviations and corresponding values:

### **Clinical Characteristics:**

1. **Age**: age at time of RRSO
2. **BMI**: body mass index
3. **Age of menarche**: age at menarche
4. **BRCA 1**: BRCA 1 status (Values: Yes/No)
5. **BRCA 2**: BRCA 2 status (Values: Yes/No)
6. **CA125**: preoperative serum CA125 levels (UI/ml)
7. **MatoRRSO**: menopause at time of RRSO (Values: Yes/No)
8. **Pregnancy nftd**: number of pregnancies normal full-term delivery
9. **Estroprogestin use**: use of estroprogestins (Values: Yes/No)
10. **History of endometriosis**: history of endometriosis (Values: Yes/No)
11. **PAPS**: history of previous abdominal/pelvic surgery (Values: Yes/No)
12. **OC FDR**: ovarian cancer status in first-degree relatives (Values: Yes/No)
13. **OC Nfdr**: number of ovarian cancer first-degree relatives
14. **OC SDR**: ovarian cancer status in second-degree relatives (Values: Yes/No)
15. **OC Nsdr**: number of ovarian cancer second-degree relatives
16. **Previous BC**: history of previous breast cancer (Values: Yes/No)
17. **BC FDR**: breast cancer status in first-degree relatives (Values: Yes/No)
18. **BC Nfdr**: number of breast cancer first-degree relatives
19. **BC SDR**: breast cancer status in second-degree relatives (Values: Yes/No)
20. **BC Nsdr**: number of breast cancer second-degree relatives

### **Breast cancer histological Features:**

1. **PR**: progesterone receptor status (Values: None, Negative, Positive)
2. **ER**: estrogen receptor status (Values: None, Negative, Positive)
3. **HER2**: human epidermal growth factor receptor 2 status (Values: None, Negative, Positive, Uncertain)
4. **Grade**: tumor grading (Values: None, G1, G2, G3)
5. **IDC**: invasive ductal carcinoma (Values: Yes/No)
6. **ISDC**: in situ ductal carcinoma (Values: Yes/No)
7. **ILC**: invasive lobular carcinoma (Values: Yes/No)
8. **IPC**: invasive papillary carcinoma (Values: Yes/No)
9. **NSIC**: not specific invasive carcinoma (Values: Yes/No)
10. **ADLI**: atypical ductal and lobular hyperplasia (Values: Yes/No)
11. **TC**: tubular carcinoma (Values: Yes/No)
