# Supplementary material for: Validation of machine learning-based models to predict and explain the risk of ovarian cancer: a multicentric study on BRCA-mutated patients undergoing risk-reducing salpingo-oophorectomy
Source: Front Oncol. 2025 Apr 15;15:1574037. doi: 10.3389/fonc.2025.1574037 (PMC12037974; doi:10.3389/fonc.2025.1574037)
Supplement: Supplementary Table 1 — Clinical characteristics of both Investigational Cohort (IC) and External Validation cohort (EVC). For categorical variables, absolute and percentage counts are reported in round brackets. For continuous variables, the median value and first (q1) and third (q3) quartiles of the distribution are indicated in squared brackets. The number of missing values (NA) is also specified. [file Table1.docx]

| **Features** | **IC** | **EVC** | **Features** | **IC** | **EVC** |
| --- | --- | --- | --- | --- | --- |
| **Overall (rare class)** | 550; 100% | 144; 100 % | **ER** |  |  |
| **Age** |  |  | None | 213 (38.7) | 48 (33.3) |
| Median; [$q_{1},$ $q_{3}$] | 49 [43; 55] | 49 [44; 55] | Negative | 129 (23.5) | 30 (20.8) |
| NA (n.; %) | 3 (0.54) | 1 (0.69) | Positive | 71 (12.9) | 32 (22.2) |
| **BMI** |  |  | NA | 137 (24.9) | 34 (23.7) |
| Median;  [$q_{1},$ $q_{3}$] | 23.4  [20.8, 26.9] | 24.5  [22.1; 27.3] | **HER2** |  |  |
| NA (n.; %) | 31 (5.6) | 17 (11.8) | None | 243 (44.2) | 53 (36.8) |
| **A. of menarche** |  |  | Negative | 128 (23.3) | 41 (28.5) |
| Median; [$q_{1},$ $q_{3}$] | 12 [12; 13] | 12 [11;13] | Positive | 29 (5.4) | 10 (6.9) |
| **BRCA 1** |  |  | Uncertain | 1 (0.18) | 2 (1.39) |
| No (n.; %) | 264 (48.0) | 66 (45.8) | NA | 149 (27.1) | 38 (26.4) |
| Yes (n.; %) | 286 (52.0) | 78 (54.2) | **Grade** |  |  |
| **BRCA 2** |  |  | None | 150 (27.3) | 53 (36.8) |
| No (n.; %) | 294 (54.5) | 68 (47.2) | G1 | 3 (0.55) | 2 (1.39) |
| Yes (n.; %) | 256 (44.5) | 76 (52.8) | G2 | 77 (14.0) | 15 (10.4) |
| **CA125** |  |  | G3 | 140 (25.5) | 42 (29.2) |
| Median; [$q_{1},$ $q_{3}$] | 10.0 [7.0; 14.3] | 10.2 [7.2; 14.5] | NA | 180 (32.7) | 32 (22.2) |
| NA (n.,%) | 257 (46.7) | 61 (42.3) | **BC FDR** |  |  |
| **Pregnancy nftd** |  |  | No (n.; %) | 310 (56.4) | 77 (53.5) |
| Median; [$q_{1},$ $q_{3}$] | 2 [1;2] | 2 [1;2] | Yes (n.; %) | 225 (40.9) | 53 (36.8) |
| NA (n.; %) | 11 (2.0) | 10 (6.9) | NA (n.; %) | 15 (2.7) | 14 (9.7) |
| **MatoRRSO** |  |  | **BC SDR** |  |  |
| No (n.; %) | 230 (41.8) | 61 (42.3) | No (n.; %) | 260 (47.3) | 66 (45.8) |
| Yes (n.; %) | 302 (54.9) | 76 (52.8) | Yes (n.; %) | 275 (50.0) | 62 (43.1) |
| NA (n.; %) | 18 (3.29) | 7 (4.9) | NA (n.; %) | 15 (2.7) | 16 (11.1) |
| **Estroprogestin use** |  |  | **BC Nfdr** |  |  |
| No (n.; %) | 268 (48.7) | 100 (69.4) | Median; [$q_{1},$ $q_{3}$] | 0 [0;1] | 0 [0;1] |
| Yes (n.; %) | 231 (42.0) | 38 (26.4) | NA (n.; %) | 15 (2.7) | 36 (25) |
| NA | 51 (9.3) | 6 (4.2) | **BC Nsdr** |  |  |
| **H.endometriosis** |  |  | Median; [$q_{1},$ $q_{3}$] | 1 [0;1] | 1 [0;1] |
| No (n.; %) | 535 (97.3) | 138 (95.8) | NA (n.; %) | 15 (2.7) | 36 (25) |
| Yes (n.; %) | 14 (2.6) | 5 (3.4) | **IDC** |  |  |
| NA | 1 (0.18) | 1(0.69) | No | 210 (38.2) | 53 (36.8) |
| **PAPS** |  |  | Yes | 209 (38.0) | 60 (41.7) |
| No (n.; %) | 310 (56.4) | 97 (67.4) | NA | 131 (23.8) | 31 (21.5) |
| Yes (n.; %) | 237 (43.1) | 36 (25.0) | **ISDC** |  |  |
| NA (n.; %) | 3 (0.50) | 11 (7.6) | No | 404 (73.5) | 105 (72.9) |
| **OC FDR** |  |  | Yes | 15 (2.7) | 8 (5.6) |
| No (n.; %) | 386 (66.9) | 105 (72.9) | NA | 131 (23.8) | 31 (21.5) |
| Yes (n.; %) | 149 (30.4) | 24 (16.7) | **NSIC** |  |  |
| NA (n.; %) | 15 (2.7) | 15 (10.4) | No | 386 (70.2) | 109 (75.7) |
| **OC SDR** |  |  | Yes | 33 (6.0) | 4 (2.8) |
| No (n.; %) | 420 (76.4) | 104 (72.2) | NA | 131 (23.8) | 31 (21.5) |
| Yes (n.; %) | 115 (20.9) | 24 (16.7) | **ILC** |  |  |
| NA (n.; %) | 15 (2.7) | 16 (11.1) | No | 399 (72.6) | 110 (69.4) |
| **OC Nfdr** |  |  | Yes | 20 (36.4) | 3 (9.1) |
| Median; [$q_{1},$ $q_{3}$] | 0 [0;1] | 0 [0;1] | NA | 131 (23.8) | 31 (21.5) |
| NA (n.; %) | 15 (2.7) | 54 (37.5) | **IPC** |  |  |
| **OC Nsdr** |  |  | No | 417 (75.8) | 112 (77.8) |
| Median; [$q_{1},$ $q_{3}$] | 0 [0;0] | 0 [0;1] | Yes | 2 (0.36) | 1 (0.69) |
| NA (n.; %) | 15 (2.7) | 57 (39.6) | NA | 131 (23.8) | 31(21.5) |
| **Previous BC** |  |  | **ADLI** |  |  |
| No (n.; %) | 211 (38.4) | 68 (47.2) | No | 419 (76.2) | 112 (77.8) |
| Yes (n.; %) | 339 (61.6) | 76 (52.8) | Yes | 0 (0) | 1 (0.69) |
| **PR** |  |  | NA | 131 (23.8) | 31 (21.5) |
| None | 231 (42.0) | 49 (34.0) | **TC** |  |  |
| Negative | 118 (21.5) | 33 (22.9) | No | 419 (76.2) | 112 (77.8) |
| Positive | 64 (11.6) | 28 (19.5) | Yes | 0 (0) | 1 (0.69) |
| NA | 137 (24.9) | 34 (23.6) | NA | 131 (23.8) | 31 (21.5) |
|  |  | *(Continued)* |  |  |  |
