# Supplementary material for: Validation of machine learning-based models to predict and explain the risk of ovarian cancer: a multicentric study on BRCA-mutated patients undergoing risk-reducing salpingo-oophorectomy
Source: Front Oncol. 2025 Apr 15;15:1574037. doi: 10.3389/fonc.2025.1574037 (PMC12037974; doi:10.3389/fonc.2025.1574037)
Supplement: Supplementary Table 2 — Summary of p-Values from Statistical Tests Comparing Clinical Data of the Investigational Cohort (IC) and External Validation Cohort (EVC). A result was considered statistically significant when the p-value was less than 0.05. [file Table2.docx]

| **Feature** | **p-value** |
| --- | --- |
| **Age** | 0.5291 |
| **BMI** | 2.38x10^(-09) |
| **A. of menarche** | 0.0123 |
| **BRCA 1** | 0.6430 |
| **BRCA 2** | 0.8848 |
| **CA125** | 0.8619 |
| **MatoRRSO** | 0.7855 |
| **Pregnancy nftd** | 0.2345 |
| **Estroprogestin use** | 0.0021 |
| **H. endometriosis** | 0.5373 |
| **PAPS** | 0.0014 |
| **OC FDR** | 0.0318 |
| **OC Nfdr** | 0.8188 |
| **OC SDR** | 0.4931 |
| **OC Nsdr** | 0.2890 |
| **Previous BC** | 0.0536 |
| **BC FDR** | 0.7896 |
| **BC Nfdr** | 0.1759 |
| **BC SDR** | 0.5468 |
| **BC Nsdr** | 0.6908 |
| **PR** | 0.1346 |
| **ER** | 0.0432 |
| **HER2** | 0.2435 |
| **Grade** | 0.2508 |
| **IDC** | 0.5588 |
| **ISDC** | 0.1045 |
| **ILC** | 0.3258 |
| **TC** | 0 |
| **IPC** | 0.6076 |
| **NSIC** | 0.1078 |
| **ADLI** | 0 |
