# Supplementary material for: Validation of machine learning-based models to predict and explain the risk of ovarian cancer: a multicentric study on BRCA-mutated patients undergoing risk-reducing salpingo-oophorectomy
Source: Front Oncol. 2025 Apr 15;15:1574037. doi: 10.3389/fonc.2025.1574037 (PMC12037974; doi:10.3389/fonc.2025.1574037)
Supplement: Supplementary Table 3 — Feature selection for (A) AllCatModel and (B) CatModel on the training set. Frequency of the features in all the diverse ratio models have been reported. Frequencies greater than 40% are highlighted in bold. The abbreviations of all the features are summarized in Supplementary File 1 . [file Table3.docx]

|  | **Frequency (%)** | | | | | |
| --- | --- | --- | --- | --- | --- | --- |
|  | **Model ratio 1:1** | | **Model ratio 1:2** | | **Model ratio 1:3** | |
| **Features** | **AllCatModel** | **CatModel** | **AllCatModel** | **CatModel** | **AllCatModel** | **CatModel** |
| Age | **78** | **78** | **85** | **85** | **89** | **89** |
| BMI | 35 | 29 | 30 | 23 | 24 | 20 |
| Age of menarche | 39 | **40** | 32 | 33 | 37 | 25 |
| BRCA 1 | **49** | **55** | 34 | **42** | 38 | **44** |
| BRCA 2 | **52** | **56** | **45** | **48** | **50** | **51** |
| CA125 | **55** | **52** | **57** | **56** | **58** | **60** |
| MatoRRSO | **62** | **62** | **65** | **65** | **76** | **76** |
| Pregnancy nftd | **50** | **52** | **59** | **57** | **60** | **56** |
| Estroprogestin use | **55** | **56** | **51** | **49** | **42** | 38 |
| History of endometriosis | **56** | **58** | 38 | **41** | 25 | 35 |
| PAPS | **43** | **45** | **44** | **46** | **40** | **42** |
| OC FDR | **56** | **51** | **64** | **58** | **65** | **63** |
| OC Nfdr | **56** | **54** | **57** | **56** | **63** | **65** |
| OC SDR | **56** | 32 | 29 | 36 | 17 | 38 |
| OC Nsdr | **50** | **53** | **49** | **52** | **46** | **48** |
| Previous BC | **45** | 37 | **63** | **58** | **76** | **65** |
| BC FDR | 37 | 34 | 35 | 31 | 30 | 27 |
| BC Nfdr | **58** | **58** | **55** | **55** | **53** | **53** |
| BC SDR | **56** | 38 | **64** | 34 | **65** | 34 |
| BC Nsdr | 32 | 30 | 33 | 29 | 28 | 27 |
| PR | **40** | - | **43** | - | **47** | - |
| ER | 35 | - | 33 | - | 37 | - |
| HER2 | **42** | - | **44** | - | **52** | - |
| Grade | **56** | - | **72** | - | **83** | - |
| IDC | **52** | - | **62** | - | **66** | - |
| ISDC | **50** | - | 37 | - | 33 | - |
| ILC | **55** | - | **54** | - | **45** | - |
| IPC | **42** | - | **37** | - | **37** | - |
| NSIC | **51** | - | **43** | - | **37** | - |
| ADLI | 30 | - | 26 | - | 22 | - |
| TC | 33 | - | 37 | - | 35 | - |
