# Supplementary material for: Validation of machine learning-based models to predict and explain the risk of ovarian cancer: a multicentric study on BRCA-mutated patients undergoing risk-reducing salpingo-oophorectomy
Source: Front Oncol. 2025 Apr 15;15:1574037. doi: 10.3389/fonc.2025.1574037 (PMC12037974; doi:10.3389/fonc.2025.1574037)
Supplement: Supplementary Table 4 — Overview on the optimal feature set. Features selected with frequency higher or equal than 40% for all the model ratios were reported in each column. [file Table4.docx]

| **AllCatModel** | **CatModel** | **Previous published model** |
| --- | --- | --- |
| MatoRRSO | MatoRRSO | Age |
| OCFDR | OCFDR | BMI |
| Estroprogestinuse | PAPS | CA125 |
| PreviousBC | BRCA1 | Age of Menarche |
| IDC | BRCA2 | BCNsdr |
| ILC | BCNfdr | Pregnancynftd |
| Grade | OCNfdr |  |
| HER2 | OCNsdr |  |
| BRCA2 | Pregnancynftd |  |
| BCNfdr | CA125 |  |
| OCNfdr | Age |  |
| OCNsdr |  |  |
| Pregnancynftd |  |  |
| CA125 |  |  |
| Age |  |  |
